# Current treatment of non‐alcoholic fatty liver disease

**DOI:** 10.1111/joim.13531

**Published:** 2022-07-07

**Authors:** Rafael Paternostro, Michael Trauner

**Affiliations:** ^1^ Division of Gastroenterology and Hepatology, Department of Internal Medicine III Medical University of Vienna Vienna Austria

**Keywords:** cirrhosis, fibrosis, non‐alcoholic fatty liver disease, non‐alcoholic steatohepatitis

## Abstract

Non‐alcoholic fatty liver disease (NAFLD) comprises a wide spectrum of pathologies ranging from non‐alcoholic fatty liver (NAFL), characterized by simple steatosis without inflammation, to non‐alcoholic steatohepatitis (NASH), characterized by steatosis of the liver accompanied by inflammation and hepatocyte ballooning, which can lead to advanced fibrosis, cirrhosis and hepatocellular carcinoma. Apart from lifestyle modifications such as weight loss, a Mediterranean diet and physical activity, only a few NAFLD‐specific pharmacological treatment options such as Vitamin E and Pioglitazone are considered by current international guidelines. However, recently randomized controlled trials with GLP‐1 agonists, FXR and PPAR ligands as well as other agents have been published and may expand the therapeutic armamentarium for NAFLD in the near future. Finally, knowledge about treating complications of end‐stage liver disease due to NASH becomes an increasingly important cornerstone in the treatment of the broad disease spectrum of NAFLD. In this review, we summarize currently available and future treatment options for patients with NAFLD that may help internal medicine specialists treat the complete clinical spectrum of this highly prevalent liver disease.

## Graphical abstract

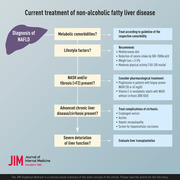

## Introduction—Definition, diagnosis and clinical staging of NAFLD

Non‐alcoholic fatty liver disease (NAFLD) is the liver disease epidemic of the 21st century, since prevalence rates range between 23% and 32% depending on the geographical region [1, 2] with numbers predicted to rise further globally. The term NAFLD itself summarizes a broad disease spectrum: non‐alcoholic fatty liver (NAFL), which is characterized by simple steatosis but absent inflammation or hepatocyte ballooning, represents the mildest manifestation. Non‐alcoholic steatohepatitis (NASH), however, is characterized by not only steatosis of the liver but also inflammation and hepatocyte ballooning, and is a more severe presentation of the disease spectrum which may lead to advanced fibrosis or even cirrhosis. In approximately 5% [3, 4] of patients [5, 6] with NAFLD complications of cirrhosis and/or hepatocellular carcinoma may occur during long‐term follow‐up. Of note, however, most patients with non‐advanced NAFLD (i.e. Fibrosis Stage 0–2) primarily show extrahepatic events during follow‐up and the predominant cause of death in these patients derives from cardiovascular disease rather than from liver‐related events [7, 8]. Apart from the importance of distinguishing between a diagnosis of NAFL or NASH and grading of disease activity, presence and stage of fibrosis need to be determined in every patient since it has been shown that prognosis is mostly influenced by the grade of fibrosis rather than presence/absence of NASH [9, 10].

Suspicion of NAFLD should be raised in patients presenting with either elevated liver enzymes (i.e. liver transaminases and/or gamma‐glutamyltransferase) or those who show hepatic steatosis on abdominal ultrasound [5]. Most importantly before diagnosing NAFLD the most common other etiologies of chronic liver disease (i.e. hepatitis, autoimmune, hereditary or cholestatic) and especially relevant alcohol consumption (≥30 g/day in men, ≥20 g/day in women) should be excluded [5]. Typically, patients presenting with one‐ or more components of the metabolic syndrome are at high risk for developing NAFLD and hepatic steatosis on imaging and/or elevated liver enzymes should raise the suspicion for NAFLD [5, 6].

A wide spectrum of non‐invasive diagnostic methods have been developed and clinically tested over the last years, the most important‐ and tested ones being vibration‐controlled transient elastography (VCTE) and non‐invasive fibrosis tests (i.e. NAFLD Fibrosis Score or FIB‐4 Score). However, non‐invasive diagnostic algorithms and risk stratification for NAFLD are out of the scope of this article but have been reviewed elsewhere [4, 11, 12].

Still, the gold standard for diagnosing, grading and staging NAFLD is liver biopsy, either percutaneous (i.e. mostly in patients without advanced chronic liver disease) or via the transjugular route (i.e. in patients with advanced chronic liver disease, severe thrombocytopenia or severe coagulopathy). Both procedures are safe with very low risk of complications [13, 14, 15]. A diagnosis of NASH is currently not possible without liver histology, however, liver biopsy is usually only performed in patients with a high pre‐test probability for advanced fibrosis and cirrhosis, as indicated by non‐invasive fibrosis tests (i.e. VCTE, non‐invasive fibrosis scores) [5, 6]. Therefore in daily clinical practice, outside clinical trials, only rather indeterminate/unclear cases regarding fibrosis stage or etiology require liver biopsy while when cirrhosis/ACLD is evident clinicians should directly proceed to HCC screening and management of portal hypertension [16]. However, even in cirrhotic/ACLD patients, other causes of liver disease should be carefully ruled out before the definitive diagnosis of NAFLD‐associated ACLD can be made [16].

**Fig. 1:**
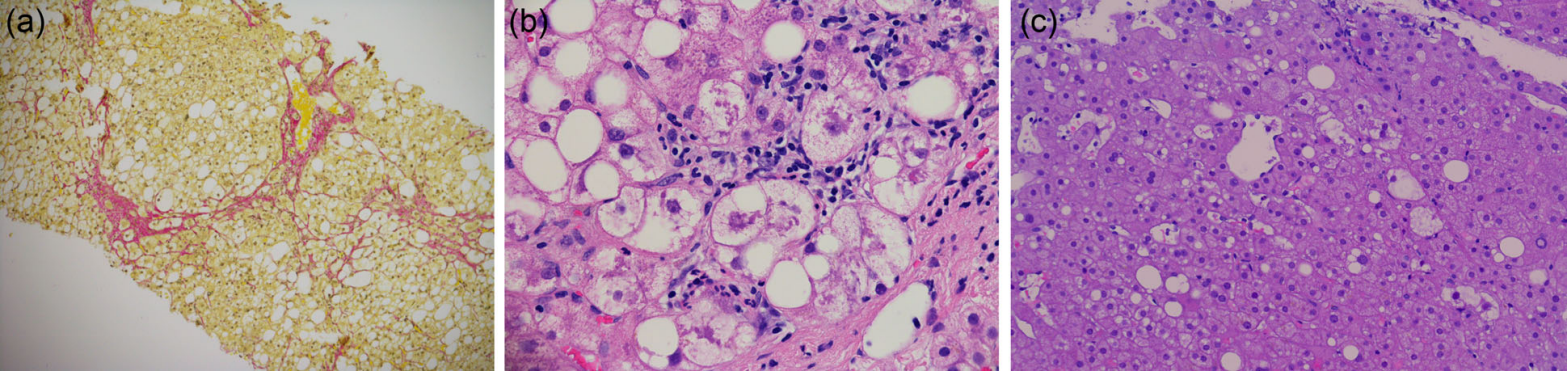
Histological features of patients with NAFLD/NASH. (a) Collagen staining with subtle bridging fibrosis—NASH CRN fibrosis score 3. (b) HE staining with classical histological landmarks of steatohepatitis: macrovesicular steatosis, lobular inflammation and hepatocyte ballooning. (c) HE staining with mild steatosis and sporadic hepatocyte ballooning. Histological slides by courtesy of Dr.med. Behrang Mozayani, FRCPath, Department of Pathology, Medical University of Vienna.

Nevertheless, once biopsy specimens are obtained, pathologists should report the grades of hepatic steatosis [reported as a percentage of lipid‐containing hepatocytes mild (Grade 1: 5–33%), moderate (Grade 2: 34–66%), severe steatosis (Grade 3: >66%)], [17] hepatocyte ballooning [absent (0), rare (1), or prominent(2)] and necro‐inflammatory activity [absent (0), mild (1), moderate (2), or severe (3)]. Finally, the NAFLD activity score (NAS) [18, 19] should be reported as the sum of the three characteristics (steatosis, ballooning, inflammation) and ranges between 0 and 8 points; however, NAFLD per se is defined by the presence of steatosis so usually, a minimum of 1 point (for steatosis) should be reported to establish a NAFLD diagnosis. Most importantly, however, it needs to be emphasized that a diagnosis of NASH should not be made based on NAS alone and rather based on evaluation of patterns as well as individual lesions (overall “gestalt”) on liver biopsies [5, 18, 19] Typical histological features of NAFLD/NASH can be seen in Fig. 1.

Another similar, but still essentially different, histologic scoring system for NAFLD is the SAF score which was developed by Bedossa and colleagues in 2012 [20]. It includes three variables Steatosis (S; 0 to 3 points available—0 points rule out NAFLD), Activity (A; Ballooning: 0 to 2 points available and Lobular Inflammation: 0 to 2 points available) and Fibrosis (F). To diagnose NASH steatosis, ballooning and lobular inflammation are all mandatory [20], which is by some seen as the more accurate way to diagnose NASH compared to proposed NAS cut‐offs, which were per se not designed to diagnose NASH but rather grade/Stage disease severity.

Liver fibrosis should be staged on a five‐point scale: no fibrosis (stage 0), pericellular fibrosis (stage 1), pericellular and portal fibrosis (stage 2), bridging fibrosis (stage 3) or cirrhosis (stage 4) [18].

Once the diagnosis of NAFLD (i.e. NAFL or NASH) has been obtained clinicians need to evaluate what treatment options there are for the individual patient (i.e. diabetic vs. non‐diabetic, no‐mild‐moderate vs. advanced fibrosis, etc.) and should tailor possible treatment strategies accordingly. In the following paragraphs, we will review those possible treatment options, their evidence and clinical applicability. Clinically relevant bullet‐points on definitions, diagnosis and staging of NAFLD have been also summarized in Table 1.

**Table 1:** Non‐alcoholic fatty liver disease—clinically relevant bullet points

Definition?	Diagnostic gold‐standard still liver biopsy At least 5% steatosis needed for formal diagnosis Discriminate between NAFL (non‐alcoholic fatty liver) and NASH (non‐alcoholic steatohepatitis) NAFL = simple steatosis but *absent* inflammation or hepatocyte ballooning NASH = steatosis *with* inflammation and hepatocyte ballooning
Non‐invasive diagnosis?	Ultrasound → look for signs of steatosis (hyperechogenic liver‐parenchyma) Vibration controlled transient elastography (VCTE; Fibroscan^TM^) → non‐invasively evaluate fibrosis VCTE values ≥10 kPa or ≥15 kPa suspicious/indicative of advanced chronic liver disease Magnetic resonance elastography (MRE) → non‐invasively evaluate steatosis and fibrosis Laboratory based fibrosis scores (FIB‐4 or NAFLD Fibrosis Score)
Invasive diagnosis?	Liver biopsy—either percutaneously (usually patients with no clinical/laboratory signs for advanced chronic liver disease and or coagulopathy) or via the transjugular route (in patients with advanced chronic liver disease, acute liver failure or other severe coagulopathies)
How to grade/stage NAFLD?	Histology: NAFLD Activity Score (NAS)—consists of three components (Steatosis 0–3 points, Inflammation 0–3 points, Ballooning 0–2 points) NAS ≥5 → cut‐off with excellent discriminative value for the presence of definite NASH; although not per se diagnostic. Histology: SAF Score (SAF), S—Steatosis (0–3 points), A—Activity (Ballooning 0–2 points, Lobular inflammation 0–2 points) and F—Fibrosis (0‐4)— importantly steatosis, ballooning and lobular inflammation are all mandatory to diagnose NASH [20] Fibrosis: Stage 0 (none)—Stage 4 (cirrhosis) Advanced fibrosis → Stages 3 and 4 If advanced chronic liver disease present → screen for complications of portal hypertension (varices, ascites, hepatic encephalopathy) and hepatocellular carcinoma (HCC; CAVE: some HCCs might also occur in the non‐cirrhotic NAFLD liver!) and treat accordingly

## Treatment Of NAFLD—Lifestyle factors, metabolic comorbidities and NAFLD‐specific therapies

According to current guidelines [5] pharmacotherapy in NASH patients should be reserved for those with significant fibrosis (≥F2) and those with less severe disease but at high risk of disease progression (i.e. metabolic syndrome, diabetes).

**Fig. 2:**
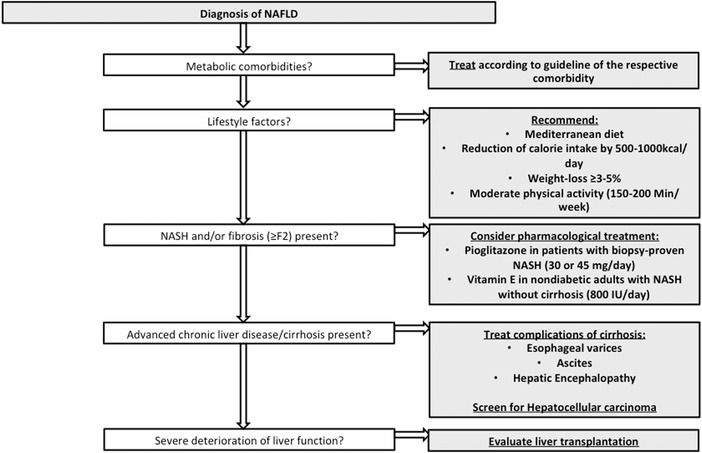
Current treatment options for NAFLD.

Nevertheless, it needs to be emphasized that once a diagnosis of NAFLD is established patients have increased overall mortality compared to non‐NAFLD patients [6, 21, 22]. However, this increased mortality mostly comes from cardiovascular‐ rather than from liver‐related outcomes; [4, 6] furthermore, cancer‐related mortality is among the leading causes of mortality in NAFLD patients, mainly driven by extrahepatic malignancies followed by hepatocellular carcinoma [23, 24]. Most importantly, once a diagnosis of NASH and/or advanced fibrosis (i.e. fibrosis stage 3 or cirrhosis) and/or portal hypertension is confirmed patients are at an increased risk for liver‐related complications (i.e. hepatic decompensation and hepatocellular carcinoma) and liver‐related mortality [9, 10, 25]. Therefore, lifestyle modifications and treatment of underlying metabolic conditions should be performed in all NAFLD patients, while specific pharmacological treatment should mainly be aimed at patients with biopsy‐proven NASH and fibrosis [6]. A short summary of a possible treatment algorithm for patients with NAFLD has been summarized in Fig. 2.

### Lifestyle factors

Diet, weight loss and physical activity are the cornerstone of every treatment for NAFLD and are recommended by both the American and European associations for the study of the liver [5, 6]. Reducing calorie intake by at least 500–1000 kcal has been shown to reduce hepatic steatosis and insulin resistance [26, 27]. Energy restriction and exclusion of NAFLD‐promoting components (i.e. processed food, products high in added fructose) are recommended by the EASL‐NAFLD guidelines [5] and generally speaking a “Mediterranean diet” should be recommended to all NAFLD patients [5].

Dieting ultimately leads to weight loss and weight loss per se has been a major link to achieving improvements in liver histology and even resolution of NASH or fibrosis. In a 12‐month lifestyle intervention program in patients with type 2 diabetes, hepatic steatosis and incident NAFLD was significantly reduced [28]. Most importantly, a study including 261 NAFLD patients with paired liver biopsies before and after lifestyle changes aiming at inducing weight loss found that a greater extent of weight loss is associated with improvement in histologic features of NASH with the highest rates of NAS reduction (100%), NASH resolution (90%) and fibrosis regression (45%) occurring in those patients with at least ≥10% of weight lost [29]. Nevertheless, it needs to be noted that only 30% of all subjects have lost at least ≥5% of their weight at week 52 (end of the study)—and this very much represents the real‐life issue of a few patients achieving weight‐loss targets. Finally, a large systemic review and meta‐analysis have shown that weight loss (≥7%) generally is safe and improves liver histology and cardiometabolic profile in NAFLD patients [30].

Regarding physical activity, current guidelines recommend 150–200 min/week of moderate‐intensity aerobic physical activities in three to five sessions [5]. Importantly, it needs to be emphasized that also in patients with advanced chronic liver disease (i.e. cirrhosis) mild‐to‐moderate exercise is safe, reduces the degree of portal hypertension and was not associated with an increased risk for variceal bleeding or other hepatic decompensation [31]. Most recently, a study investigating an intervention consisting of a hypocaloric diet and 60 min/week supervised physical activity in compensated cirrhosis with portal hypertension and a BMI >26 showed a significant decrease in the degree of portal hypertension after 16 weeks of intervention [32], with a weight‐loss of >10% being associated with an even greater decrease in portal pressure. Of note, no episode of clinical decompensation occurred during the intervention [32].

To summarize the cornerstone of every treatment in all patients with NAFLD should contain the following three components:

1. Mediterranean diet aiming to reduce the average daily calorie intake by at least 500–1000 kcal.
2. Weight loss induced by diet and physical activity aiming at losing at least 3–5% of body weight.
3. Moderate physical activity aiming at 150–200 min/week—also in patients with NAFLD‐associated advanced chronic liver disease.

### Pharmacological treatment options

Guideline‐recommended pharmacological treatment options for NAFLD patients are scarce and currently, only Vitamin E and the proliferator‐activated receptor gamma (PPAR‐y) ligand Pioglitazone are recommended for selected patients by the European‐ and American Association for the Study of the Liver [5, 6].

### Vitamin E

The anti‐oxidative effect of Vitamin E is thought to contribute to its promising results in randomized trials showing a significant improvement in NASH. In 2010, the so far largest randomized trial on Vitamin E was published (PIVENS‐Trial [33]). It included 247 adults with biopsy‐proven NASH but without diabetes and compared Vitamin E (800 IU once daily) versus Pioglitazone (30 mg once daily) versus Placebo with the primary study endpoint defined as an improvement in histologic findings (improvement by 1 or more points in a hepatocellular ballooning score; no increase in fibrosis score; and either decrease of NAS to ≤3 points or of at least ≤2 points, with at least a 1‐point decrease in either lobular inflammation or steatosis) [33]. Vitamin E treatment resulted in a significantly higher rate of NASH improvement (43% vs. 19%, *p* = 0.001) as compared with placebo. However, the grade of fibrosis did not improve [33]. Most importantly, adverse events in the Vitamin E group were not significantly different compared to Pioglitazone or placebo [33]. A study evaluating the effect of Vitamin E on clinical outcomes in 236 NASH patients with bridging fibrosis or cirrhosis found that indeed 800 IE/day decreased the risk of death or transplantation and hepatic decompensation —both in diabetic and in non‐diabetic patients [34]—and therefore adds important data into the daily clinical use of Vitamin E. Nonetheless, the latter study was no randomized controlled trial and therefore results should be interpreted cautiously. While the PIVENS trials only included non‐diabetic NASH patients, it has been shown that Vitamin E treatment alone (800 IE/day) was ineffective in reaching the primary endpoint (two‐point reduction in NAS from two different parameters, without worsening of fibrosis) in a randomized trial including 105 patients with type 2 diabetes and biopsy‐proven NASH [35]. Again no improvement in fibrosis was seen [35]. Possible side effects of Vitamin E include an increased bleeding risk, prostate cancer, heart failure and hemorrhagic stroke and those should be discussed with the patient, even though they are rarely seen [4, 6].

As of 2022 the current (2016) EASL guidelines cautiously recommend (“could be used”) Vitamin E treatment for selected patients with NASH and at least significant fibrosis (≥F2) [5] while the current practice guidance endorsed by the AASLD states that Vitamin E (800 IU/day) “may be considered” for treating non‐diabetic patients with NASH [6]. Most importantly, Vitamin E is currently *not* recommended to treat NASH in diabetic patients, NAFLD without liver biopsy, NASH cirrhosis and cryptogenic cirrhosis [6].

### Pioglitazone

Even though the PPAR‐y ligand Pioglitazone (30 mg/day) did not reach the pre‐defined primary study endpoint in the PIVENS trial, which was set at a significance level of *p* = 0.025 due to two primary comparisons, 34% in the Pioglitazone group versus 19% in the placebo group (*p* = 0.04) showed an improvement in liver histology as defined in the primary outcome [33]. Most importantly, 47% with Pioglitazone versus 21% with Placebo showed a resolution of definite NASH (*p* = 0.001) [33]. Similar to the Vitamin E treatment arm, fibrosis was not affected by Pioglitazone treatment [33]. Adverse events per se were not increased in the Pioglitazone treatment arm; importantly, however, a significant mean weight gain of +4.7 kg at week 96 was seen [33], which however could be part of the therapeutic action (lipid partitioning with the expansion of subcutaneous adipose tissue) [36]. While all diabetic patients were excluded from the PIVENS Trial, a randomized controlled trial including 101 patients with either pre‐ or type 2 diabetes found that 51% in the Pioglitazone group (45 mg/day) had resolution of NASH and 58% achieved the primary outcome of the study (reduction of ≥2 NAS points in two histologic categories without worsening of fibrosis), both significantly [37]. Interestingly, in their study, Pioglitazone treatment was also associated with a significant improvement in fibrosis score. However, weight gain was also significantly higher in the treatment group [37].

Nevertheless, it seems that a significant reduction in fibrosis score under Pioglitazone treatment is only seen in type 2 diabetic patients since Bril et al. showed a significant reduction of fibrosis with 45 mg/day. Pioglitazone treatment was only seen in type 2 diabetic patients, not in those with prediabetes [38]. While the dosage in this study was higher than in the PIVENS Trial (45 mg/day vs. 30 mg/day) duration of therapy was shorter and one could argue that similar results could have been seen in non‐diabetic patients if the study drug dose was higher.

Positive side effects of Pioglitazone treatment being improvement of insulin sensitivity and diabetic control should be weighed against its negative side effects including weight gain, fluid retention, bone loss and a possible increase in bladder cancer [4, 6]. However, as long as weight gain is not due to fluid retention it may be due to induction of a healthy obese phenotype and therefore could be clinically acceptable [36]. Most importantly, Pioglitazone is contraindicated in patients with NYHA class III or IV heart failure [4].

Finally, the current EASL guidelines [5] state that Pioglitazone “could be used” for the treatment of patients with NASH and significant fibrosis, while the AASLD suggests that it “may be used” for treating biopsy‐proven NASH patients with and without a type 2 diabetes [6].

### Other pharmacological treatment options

Apart from Vitamin E and Pioglitazone, several trials testing mechanistically different types of medication in NAFLD have been published throughout the last few years and have shown promising results. However, none have yet made their way into national‐ or international guidelines. Nevertheless, we will outline the most important clinical findings in the following chapters, stratified by pharmacological mechanisms of action, while a detailed review of emerging therapeutic targets for NAFLD can be found elsewhere [39].

### GLP‐1 agonists

The glucagon‐like peptide‐1 receptor agonist semaglutide has shown a significantly higher percentage of patients with NASH resolution (and no worsening of fibrosis) compared to placebo in a 72‐week, double‐blind phase 2 trial involving 320 patients with biopsy‐confirmed NASH and fibrosis stage 1–3^40^. Improvement in fibrosis stage was seen in 43% of NASH patients and 33% of placebo patients, but this difference was not statistically significant [40]. Importantly, around 38% of patients in the study had no (!) diabetes mellitus, however all had at least a BMI >25. Also of note, the semaglutide dosage used (0.1, 0.2 or 0.4 mg once‐daily) was significantly higher than in its main indication (treatment of diabetes mellitus type II). A previous study investigating the efficacy of the GLP‐1 agonist liraglutide in 52 overweight patients with clinical evidence of NASH showed a significantly higher rate of NASH resolution in the liraglutide group compared to placebo [41]. Most importantly, 9% in the liraglutide group versus 36% in the placebo (*p* = 0.04) group showed a progression of fibrosis [41]. A recent meta‐analysis consisting of 11 RCTs that investigated GLP‐1 agonists in NAFLD patients concluded that their overall clinical effect lies mainly in NASH resolution rather than fibrosis improvement [42].

Thus current guidelines do not recommend GLP‐1 agonists for patients with NAFLD outside their labeled indications (treatment of diabetes mellitus and/or obesity).

Recent data suggested possible positive effects of dual GLP‐1/Glucagone or GLP‐1/GIP Receptor ligands [43, 44] and those might be promising future targets, although further studies are needed to prove their clinical efficacy.

### DPP‐IV inhibitors and SGLT2 inhibitors

Studies investigating the effect of DPP‐IV inhibitors have all shown disappointing results and therefore DPP‐IV inhibitor treatment is not recommended for NAFLD patients outside their labeled indications [5, 6].

However, studies investigating sodium‐glucose cotransporter protein 2 (SGLT2) inhibitors have consistently shown a reduction in liver transaminases and improvement of imaging‐based biomarkers [45] and, therefore, might be a treatment option not only in diabetic NAFLD patients but also in those without diabetes, although large randomized trials are still needed to confirm this assumption.

### FXR ligands

In the FLINT trial [46], the effect of the steroidal farnesoid X nuclear receptor (FXR) ligand obeticholic acid (25 mg/daily) was tested in a 72‐week randomized trial involving 283 patients with non‐cirrhotic biopsy‐proven NASH. Significantly more patients in the obeticholic acid arm (45%) versus placebo (21%) showed improved liver histology [46] (defined as decrease in NAS ≥2 points without worsening of fibrosis). Nevertheless, while the primary endpoint was reached, no statistically significant effect on the resolution of NASH was seen, which could limit direct clinical usefulness. Importantly however obeticholic acid improved fibrosis in 35% of patients versus only 19% in the placebo arm (*p* = 0.004) [46]. Pruritus was the main side effect of obeticholic acid (33% vs. 6% placebo) [46]. In 2019 interim data from the REGENERATE trial [47], including 1968 patients with biopsy‐proven NASH and fibrosis stages F2‐3 or F2 with at least one accompanying comorbidity, with 931 patients included in the interim analysis was published [47]. Primary endpoints for the 18‐month interim analysis were fibrosis improvement (≥1 stage) with no worsening of NASH or NASH resolution without worsening of fibrosis [47]. Improvement in fibrosis was seen in 12% of the placebo group, 18% with obeticholic acid 10 mg (*p* = 0.045) and 23% in the obeticholic acid 25 mg (*p* = 0.0002) group. However, the proportion of NASH resolution was not significant between the groups [47]. Similar to the previous study, pruritus was the most common adverse event.

Both studies, FLINT and REGENERATE, however also showed an unfavorable effect on patients' lipid profile, that is, decrease in HDL and increase in LDL and this should be cautiously monitored in NAFLD patients under FXR ligand therapy.

Results were also published regarding monotherapy with non‐steroidal FXR agonists such as cilofexor [48, 49] and tropifexor [50] where the primary endpoint was not met in both studies. The ATLAS trial however tested a combination therapy of a non‐steroidal FXR agonist (cilofexor) with a lipogenesis inhibitor (firsocostat) and found a significant improvement of NAS subcomponents (steatosis, lobular inflammation and ballooning); however, there were no effects on fibrosis [49].

In summary, FXR ligands have shown first promising results in the RCTs investigating their clinical efficacy. Nevertheless, open questions regarding optimal dosing to minimize the potentially deleterious side effects of dyslipidemia and pruritus and the pathophysiological mechanisms behind those side effects are still unanswered and warrant further research [51].

### FGF19 mimetics

Recently published data investigating the effects of Aldafermin, an analogon of the FXR‐regulated Fibroblast‐Growth‐Factor 19 (FGF19), in patients with NASH and fibrosis stage 2 or 3 did not show improvement of fibrosis or resolution of NASH after 6 months of therapy, while improved hepatic fat content measured via MRI‐PDFF was seen [52]. However, due to the rather short time of therapy (6 months) results of ongoing long‐term studies (ALPINE) are eagerly awaited.

### FGF‐21 mimetics

Pegbelfermin showed a reduction in hepatic fat (measured via MRI‐PDFF) and liver transaminases over a 16‐week treatment period as well as an improved lipid profile; [53] however, no histological readouts were available which hampers applicability of the results and warrants further studies on this compound.

A Phase IIa study showed promising results (48% fibrosis improvement ≥1 stage; 28% both NASH resolution and fibrosis improvement) for the FGF‐21 mimetic efruxifermin [54] that calls for Phase IIb trials.

### PPAR agonists

Apart from the PPARγ agonist Pioglitazone which has found its way into international guidelines, several studies have reported data on the effects of PPAR‐δ, ‐α/δ, ‐ α/γ and most recently Pan‐PPAR agonists.

The PPARδ agonist seladelpar has shown an improvement in liver enzymes however without changes in hepatic fat (measured via MRI‐PDFF) [55], no full manuscript has yet been published.

Elafibranor, a PPARα/δ agonist, has not met the primary endpoint (NASH Resolution) in the large Phase III RESOLVE‐IT Trial [56].

Two Phase II trials have investigated the effects of saroglitazar, a PPARα/γ agonist, and found improvement of ALT and hepatic fat (measured via MRI‐PDFF) [57] but no improvement of NAS (primary endpoint: delta change of NAS from baseline to Week 24 biopsy) [58].

Finally and most recently the Pan‐PPAR agonist Lanifibranor reached the primary endpoint of a decrease in SAF‐A score of at least two points in a large Phase 2b Trial [59]—a dose‐dependent effect was seen with more patients achieving the primary endpoint with 1200 mg versus 800 mg. Most importantly, resolution of NASH without worsening of fibrosis (49% with 1200 mg Lanifibranor, vs. 39% with 800 mg vs. 22% Placebo), improvement in fibrosis of at least one stage without worsening of NASH (48% vs. 34% vs. 22%) and resolution of NASH plus improvement in fibrosis stage of at least 1 (35% vs. 25% vs. 9%) all favored the study drug as compared to placebo. Diarrhea, nausea, peripheral edema, anemia and weight gain were all seen more frequently in patients receiving Lanifibranor [59].

### THR‐beta agonists

The Thyroid Hormone Receptor Beta (THR‐B) Agonist Resmetirom (MGL‐3196) reduced hepatic fat content (assessed via MRI‐PDFF) after 12 and 36 weeks of treatment with positive effects on lipid profiles [60]. Here, a large Phase III trial (MAESTRO) is ongoing to evaluate the effects of Resmetirom on hard clinical endpoints defined as the resolution of NASH without worsening of fibrosis and prevention of progression to cirrhosis. The results are eagerly awaited. Another agent, VK2809, also showed an improvement in MRI‐PDFF measured liver fat content after 12 weeks of treatment in a Phase IIa trial [61]. A complete Phase II trial (VOYAGE) is currently ongoing.

### Anti‐inflammatory/anti‐fibrotic therapies

Disappointing data from studies investigating anti‐inflammatory/anti‐fibrotic effects have been published within the last years, the largest negative studies were with Selonsertib, a selective ASK‐1 inhibitor, in the STELLAR Trials [62], Cenicriviroc, a C‐C chemokine receptor type 2 and 5 dual antagonists, in the CENTAUR Study [63] and Simtuzumab, a monoclonal Lysyl oxidase‐like 2 antibody [64].

### Combination therapies

Since several studies have shown “not as good as expected” results regarding the effects of a single drug on either resolution of NASH and/or improvement of fibrosis, a very elegantly written review by Dufour JF et al. [65] has recently outlined possible promising combination therapies that could show significant results in both clinically relevant endpoints (NASH resolution, fibrosis improvement). Nevertheless, the primary endpoint of ≥1 stage improvement of fibrosis without worsening of NASH was not reached in any of the combination therapies tested in the ATLAS trial (cilofexor/firsocostat; cilofexor/selonsertib; firsocostat/selonsertib vs. placebo) [49].

Future studies investigating combination therapies are therefore eagerly awaited.

### Bariatric surgery

In morbidly obese patients with NAFLD/NASH, bariatric surgery may lead to improvement of NASH and/or even fibrosis [66]. This might be due to the high remission rates of type II diabetes after bariatric surgery where studies have shown that around 72–75% showed diabetes resolution up to 2 years after surgery [66, 67, 68]. Also, glycemic control seems to be significantly improved by bariatric surgery [66]. Additionally, the positive effects on lipid metabolism and inflammatory activity are thought to contribute to positive effects on severity of NAFLD [66]. Nevertheless, and importantly, NASH per se is currently not (yet) an established indication for bariatric surgery.

Several studies have investigated the effects of bariatric surgery on histologic results comparing pre‐ and post‐surgery liver biopsies and those have been elegantly summarized in a recent review [66]. Importantly almost all showed an improvement in all components that determine NAFLD severity: steatosis, inflammation and fibrosis. However, it needs to be emphasized that in some patients worsening of NAFLD was seen. Also, studies have shown that while NASH resolution was achieved in the majority of patients, a considerable number were still found with histologically advanced fibrosis despite NASH resolution [69]. While the end‐stage liver disease is a well‐known contraindication for bariatric surgery no study has yet shown reduced liver‐related mortality [66]. A small case‐control study has even investigated the effects of laparoscopic sleeve gastrectomy in 13 patients with cirrhosis that were matched to 26 non‐cirrhotic patients: no postoperative mortality was seen in either group and complication rates did not differ between cirrhotic versus non‐cirrhotic patients [70].

## Treatment of NAFLD‐associated advanced chronic liver disease

Advanced chronic liver disease (ACLD) can generally be suspected in patients showing high non‐invasive laboratory‐based fibrosis scores (FIB‐4 or NAFLD Fibrosis scores) or values suggestive of advanced fibrosis or cirrhosis using imaging methods such as vibration‐controlled transient elastography (VCTE) or magnetic resonance elastography (MRE). Usually, VCTE is widely available and values of >10 kPa are suggestive, while values >15 kPa are highly suggestive of ACLD [71, 72]. In the specific etiology of NAFLD, the threshold for ruling‐out/in advanced fibrosis ranges between 9.9 and 11.4 kPa in the STELLAR trials [73] while in a recently published large meta‐analysis a lower threshold of 7.4 kPa (90% Sensitivity) and upper threshold of 12.1 kPa (90% specificity) was published [74]. Higher values further increase the accuracy for non‐invasively predicting clinically significant portal hypertension (CSPH), whereas in patients with non‐obese NASH ACLD a VCTE value ≥25 kPa is sufficient to rule in CSPH [72]. Furthermore, ACLD should be suspected in all patients with NAFLD showing clinical‐, laboratory, or radiological signs of portal hypertension including ascites, hepatic encephalopathy, esophageal varices or portalhypertensive gastropathy, splenomegaly on abdominal ultrasound or laboratory alterations such as thrombocytopenia or impaired liver synthesis parameters (i.e. INR, albumin).

Once the clinical, radiological or histological diagnosis of advanced chronic liver disease/cirrhosis is made every patient should be staged according to widely known disease severity scores for cirrhosis (Child Pugh Score, MELD score), screened for the presence of esophageal or gastric varices [75, 76] and ultimately be classified as either “compensated” or “decompensated” ACLD. Most importantly, screening for hepatocellular carcinoma should be performed at least every 6 months using abdominal ultrasound, or CT/MRI in case of significant obesity, in combination with alpha‐fetoprotein (AFP) [77, 78]. Importantly though the role of HCC surveillance in patients with NAFLD without ACLD/cirrhosis is unclear and HCC may as well occur in non‐cirrhotic NAFLD livers. Therefore screening for HCC is also recommended in patients with ≤F3 after individual risk assessment [77, 79] for example, in those with pronounced metabolic syndrome.

If VCTE is available, non‐obese compensated patients with values <15 kPa and a platelet count > 150 G/L can avoid screening endoscopy [71, 76, 80, 81]. For obese compensated NAFLD patients, however, specific cut‐offs have been suggested: using the VCTE M probe (medium size probe) a cut‐off of <30 kPa and platelet count >110 G/L seems appropriate to rule out high‐risk esophageal varices [82], whereas in case the M probe delivers unreliable measurements (due to obesity) the XL probe should be used and the expanded Baveno VI criteria (VCTE <25 kPa, platelet count >110 G/L) applied [82, 83]. In case either VCTE or platelet count is out of the suggested thresholds, upper gastrointestinal endoscopy should be performed [76]. Gastroesophageal varices (GOV) should then be graded according to international standards: no varices, low‐risk GOVs (<5 mm) and high‐risk GOVs (>5 mm, Child Pugh Class C or red spot signs) [76, 84].

In case GOVs are present and patients have never experienced variceal bleeding in the past, primary prophylaxis of variceal bleeding with non‐selective beta‐blockers (NSBB; Carvedilol: Starting dose: 6.25–12.5 mg/day [85]; Propranolol: Starting dose 20–40 mg/day—titrate to a maximum dosage of 160 mg/day in patients without‐, and 80 mg/day in patients with ascites) is indicated [76, 86]. NSBB dosage should generally be increased until a target heart rate of 55–60/bpm is achieved and systolic blood pressure does not decrease below 90 mmHg [76]. If contraindications for NSBB therapy (i.e. severe asthma, COPD) exist or the patient does not tolerate the therapy, endoscopic variceal ligation of the GOVs should be applied [76]. However, if previous variceal bleeding has occurred in the past, secondary prophylaxis of varcieal bleeding including the combination of NSBB therapy and endoscopic variceal ligation is indicated [76].

NSBB response rates and efficacy in NAFLD patients have hardly been studied throughout the last years. Data from our group [87] found that 55.3% of patients with NASH cirrhosis undergoing NSBB therapy for either primary‐ or secondary prophylaxis of variceal bleeding were NSBB responders (median Propranolol dosage: 80 mg/d, median Carvedilol dosage: 12.5 mg/d). Interestingly, the presence of diabetes mellitus was associated with a reduced probability of achieving NSBB response [87]. Most importantly, in our study, those responding to NSBB therapy did not experience variceal bleeding during follow‐up.

Finally, irrespective of primary‐ or secondary prophylaxis NSBB therapy should, at least temporarily, be stopped in case a patient develops severe/refractory ascites *and* systolic blood pressure <90 mm Hg *or* acute kidney injury *or* spontaneous bacterial peritonitis *or* severe hyponatremia (<125 mmol/l) [76, 88, 89, 90].

Statins can usually safely be used in patients with NAFLD [6] and dyslipidemia and may also even counteract NASH [91]. Although, probably due to concerns about safety and statin use in chronic liver disease patients, real‐life data from the United States has shown that only 56% of NAFLD patients with at least one indication for statin therapy were actually prescribed statins [92]. Most importantly in patients under secondary prophylaxis of variceal bleeding, the addition of statin therapy to standard of care has shown a survival benefit in patients with Child‐Pugh class A or B cirrhosis; [93] however, only five patients with NAFLD have been included in this study. In regard to adverse events, no statistical difference was seen between the simvastatin and placebo arm, however, rhabdomyolysis occurred in two (2.8%) patients [93]. A meta‐analysis and retrospective cohort study has also shown a survival benefit of statin therapy in patients with ACLD [94, 95]. A recent study has however reported increased adverse events rates in patients with decompensated cirrhosis under 40 mg/day of simvastatin (combined with rifaximin) therapy, compared to 20 mg/day [96]. Current recommendations state that statin therapy may be used in patients with NASH cirrhosis; however, it should be avoided in decompensated cirrhosis [6].

Even though metformin does not play a role in the treatment of NASH, outside its classical indication in the treatment of diabetes, promising data have been published regarding positive clinical effects of metformin on prognosis (mortality, hepatic decompensation) and even HCC development [97, 98, 99, 100]. Nevertheless, Metformin use in NAFLD ACLD is not recommended outside its clinical indication, although if indicated its pleiotropic effects on clinical outcomes could be beneficial for the individual patient.

In general, cirrhotic NAFLD patients should be seen at the outpatient clinic at least every 6 months; however, in case of decompensation, those intervals should be shortened at the clinicians' discretion. Apart from treating esophageal varices, patients with NAFLD‐associated ACLD should be classified as either being “compensated‐” or “decompensated”, since any hepatic decompensation significantly impairs prognosis in cirrhotic patients [101, 102]. Hepatic decompensation per se is defined as the first occurrence of ascites, hepatic encephalopathy, variceal bleeding and jaundice [75, 102]. Portal hypertension is the leading driver of hepatic decompensation in cirrhotic patients [103], also in NAFLD‐associated ACLD (Paternostro et al. unpublished data). Finally, in case of severe deterioration of liver function (i.e. indicated by a MELD ≥15 or pronounced hepatic decompensation such as refractory ascites or failure of secondary prophylaxis of variceal bleeding), the option for liver transplantation should be discussed and the patient should be presented to a tertiary‐care liver transplant center [104]. Transjugular intrahepatic portosystemic shunt (TIPS) should be used in those patients with refractory ascites or failure of secondary prophylaxis of variceal bleeding, also as an option to bridge to transplant [76].

Finally, malnutrition [105], frailty [106, 107] and especially sarcopenia [108, 109, 110, 111] have become increasingly important in patients with advanced chronic liver disease and should be evaluated and treated in each patient. This is especially important in obese patients with NAFLD since those initially do not appear malnourished or sarcopenic; however, studies have shown high prevalence rates of sarcopenia also in NAFLD patients [112, 113]. Relevant bullet points regarding treatment of patients with NAFLD‐associated ACLD have been summarized in Table 2.

**Table 2:** NAFLD‐associated advanced chronic liver disease—treating compensated and decompensated patients

Screen for hepatocellular carcinoma every 6 months using abdominal ultrasound + alpha‐fetoprotein in all NAFLD‐ACLD patients. Screening for HCC indicated in selected patients with advanced fibrosis *or* non‐invasive markers highly suggestive of the latter—individual patient risk assessment necessary Use vibration controlled transient elastography in combination with platelet count to rule out high‐risk GOVs. If ruling out high‐risk GOVs is not possible or patient is decompensated—perform upper gastrointestinal endoscopy. If GOVs present and no prior variceal bleeding initiate primary prophylaxis of variceal bleeding using non‐selective beta‐blockers (NSBB; i.e. Carvedilol or Propranolol). If prior variceal bleeding secondary prophylaxis should be initiated using the combination of NSBBs and endoscopic variceal band ligation. Outpatient visits every 6 months in compensated patients, in case of decompensation tighter visits at the clinicians discretion indicated. Screen and treat any hepatic decompensation, the most frequent being ascites, hepatic encephalopathy and variceal bleeding. In case liver function severly deteriorates (i.e. MELD ≥15, pronounced hepatic decompensation such as refractory ascites or refractory secondary prophylaxis of variceal bleeding) present patient to tertiary care center to discuss the option for liver transplantation.

## Conclusions

NAFLD is a highly prevalent liver disease that covers a wide spectrum of clinical presentations with patients initially being at high risk for cardiovascular events, while some may progress to advanced fibrosis or even cirrhosis and are therefore at risk for hepatic decompensation and liver‐related mortality. Pharmacological treatment options for NAFLD are still limited and the cornerstone of any treatment is diet, weight loss and physical exercise. Current pharmacological treatments include Vitamin E or Pioglitazone, while large randomized trials have shown promising results for GLP‐1 agonists, FXR and PPAR ligands. Once patients develop advanced chronic liver disease (i.e. cirrhosis) management should focus on liver‐related complications such as esophageal varices and associated bleeding and prevention of hepatic decompensation such as ascites or hepatic encephalopathy. Most importantly screening for hepatocellular carcinoma should be performed in all cirrhotic patients, while it may be performed in selected patients with biopsy‐proven advanced fibrosis (F3) or where non‐invasive fibrosis markers are suggestive of advanced fibrosis.

Finally, in patients with end‐stage liver disease due to NAFLD, liver transplantation should be considered and the patient referred to a tertiary care liver transplant center.

## Funding

This work was supported by the grant F7310‐B21 from the Austrian Science Foundation (to MT).

## Conflict of interest

MT received speaker fees from Bristol‐Myers Squibb (BMS), Falk Foundation, Gilead, Intercept and Merck Sharp & Dohme (MSD); advisory board fees from Albireo, BiomX, Boehringer Ingelheim, Falk Pharma GmbH, GENFIT, Gilead, Intercept, Janssen, MSD, Novartis, Phenex, Regulus and Shire; travel grants from AbbVie, Falk, Gilead, and Intercept; and research grants from Albireo, Alnylam, CymaBay, Falk, Gilead, Intercept, MSD, Takeda and UltraGenyx. He is also a coinventor of patents on the medical use of norUDCA filed by the Medical Universities of Graz and Vienna.

## Author contributions

RP drafted the manuscript, which was then critically revised by MT.
